# Herpes zoster in an 11‐month‐old immunocompetent infant: A rare case report

**DOI:** 10.1002/ccr3.2912

**Published:** 2020-04-30

**Authors:** Chandra Mohan Sah, Sandeep Shrestha, Nagendra Chaudhary

**Affiliations:** ^1^ Department of Dermatology and Venereology Universal College of Medical Sciences Bhairahawa Nepal; ^2^ Department of Pediatrics Universal College of Medical Sciences Bhairahawa Nepal

**Keywords:** Herpes zoster, immunocompetent, infant, neonatal varicella, *Varicella‐zoster* virus

## Abstract

Herpes zoster generally occurs after the exposure of VZV following varicella. This case report emphasizes a rare case of HZ in an infant without prior history of varicella or its exposure in the baby, other siblings, and mother during or after the pregnancy.

## Graphical abstract

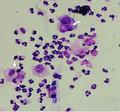

## 1 INTRODUCTION

Primary varicella infection is usually acquired in children, and reactivation occurs in elderly to produce Herpes zoster (HZ). We report a rare case of HZ in an 11‐month‐old immunocompetent infant without prior history of varicella or its exposure in the baby, other siblings, and mother during or after pregnancy.

Primary infection by *Varicella‐zoster* virus (VZV) causes varicella. These viruses remain latent within sensory neuron of dorsal root ganglion. Reactivation under certain circumstances produces segmental eruption known as herpes zoster (HZ).^1^ Disease presents with dermatomal pain or numbness followed by eruption of grouped vesicular lesions. HZ commonly occurs in elderly, but in rare circumstances, it can occur in healthy and immunocompromised children. In infant, HZ generally occurs due to the reactivation of VZV acquired in utero from maternal varicella. Rarely, reactivation may be due to early or unrecognized varicella.^2^

## 2 CASE PRESENTATION

An 11‐month‐old female child was brought to the pediatric outpatient department with complaints of fever for two days and rashes involving over the right side of trunk reaching up to the right sub costal region. She also had mild coryza.

**Figure 1:**
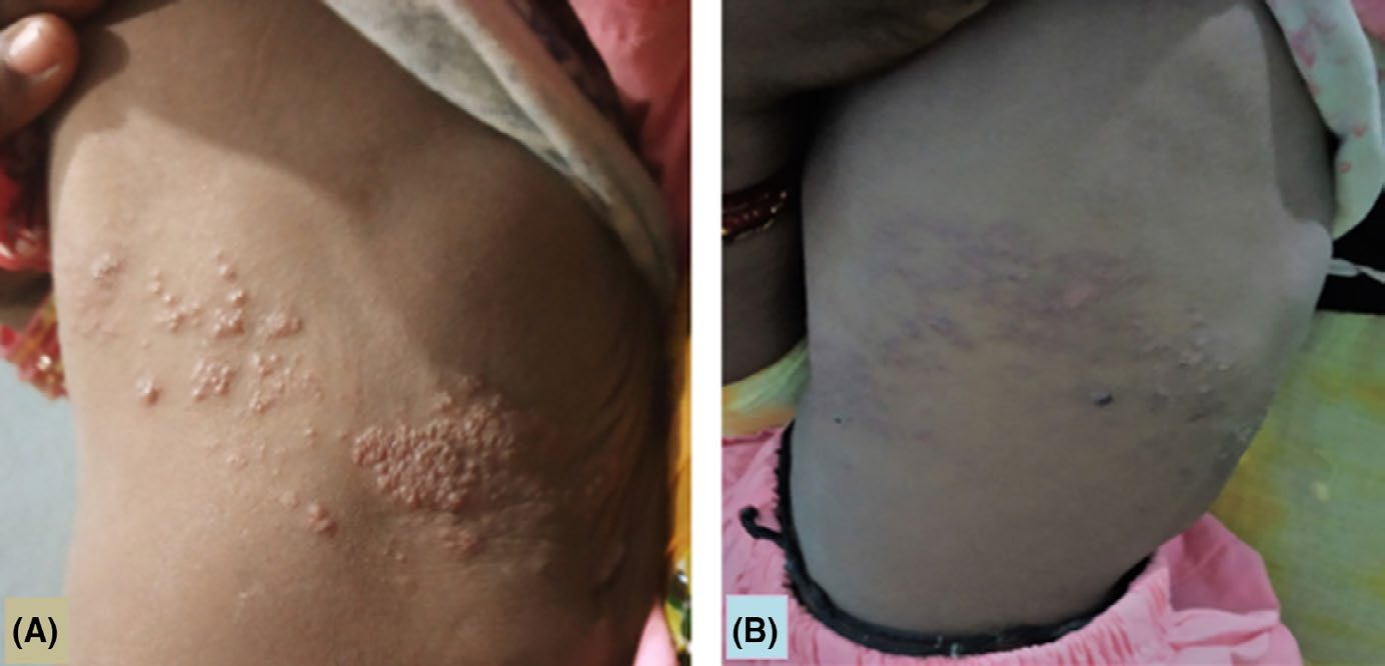
A, Showing grouped vesicles in T9 dermatome and B, showing the same lesion on 7th day of treatment

On examination, she was febrile (temperature 100.8℉), pulse rate—110/min, and respiratory rate—30/min. Her weight was 7.5 kg (10th centile). General physical examination showed pallor but no icterus, cyanosis, clubbing, edema, or lymphadenopathy. Systemic examination was normal. Skin examination showed grouped vesicles in thoracic (T9) dermatome on right side of the body (Figure 1A). Vesicles were present over erythematous base and contained clear fluid. No other abnormality was present.

There was no history of maternal varicella in the past nor during the pregnancy. Maternal TORCH profile done during pregnancy was negative. Detailed history was obtained from the family members, and none of them had any varicella‐like illnesses in the past. Parents did not give any history of varicella‐like rash in the child previously. On investigation, complete blood count showed hemoglobin—9 gm/dL, total leukocyte count—5600 cells/mm^3^, and platelet count—4.5 lakhs/mm^3^. Peripheral smear was suggestive of microcytic hypochromic anemia. Human immunodeficiency virus (HIV ELISA) screening was negative.

**Figure 2:**
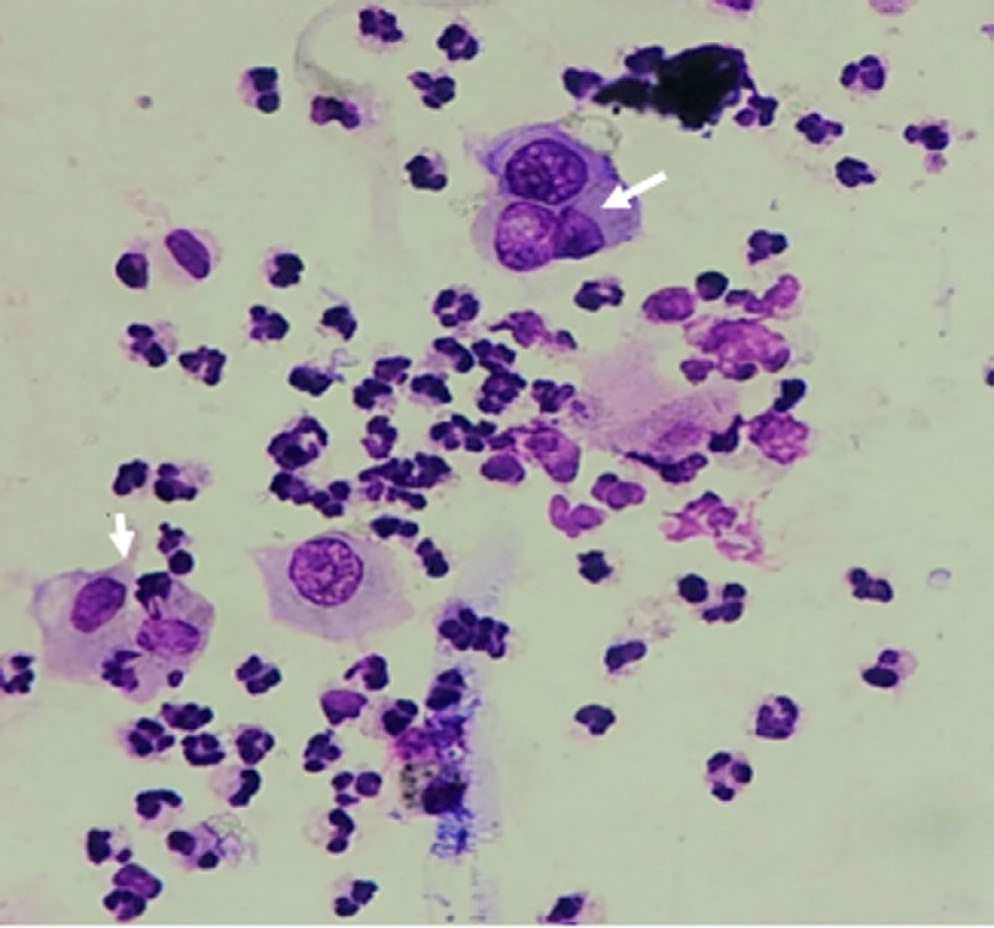
Smear showing many acantholytic cells with benign squamous cells with single, bi‐, and tri‐nucleated cells (staining—Giemsa stain; magnification—40×)

Tzanck smear from the base of the vesicle showed multinucleated giant cells which supported the diagnosis of HZ in a child with vesicular rash (Figure 2). Antibodies (IgM and IgG) against VZV were negative in the mother.

As the child presented to us before 72 hours of skin eruptions, she was treated with oral acyclovir at the dose of 20 mg/kg/dose, five times a day for seven days along with topical antibiotic (fusidic acid cream) and antipyretic (oral paracetamol). Follow‐up visit on day seven showed improvement in the skin lesions (Figure 1B). On follow‐up, nutritional counseling was given along with oral iron supplements.

## 3 DISCUSSION

Primary infection by VZV causes varicella, and reactivation produces segmental eruption known as HZ.^1^ The annual incidence of HZ in dermatology clinics of Nepal is 0.55%‐0.65% with the mean age of presentation between 34 and 40 years.^3^ VZV reactivation in infancy and childhood is rare, but can occur following infection acquired in utero and rarely during early infancy.^4^, ^5^ Reactivation is uncommon even after primary varicella infection before one year of age or in immunocompetent state.^6^ HZ of infancy differs from that of adult as it is not generally associated with pain and postherpetic neuralgia. As the child was only 11 months old, we could not inquire regarding pain and discomfort. The earliest reported case of HZ in literature was by Feldman et al in a four‐day‐old child from USA.^7^ In his case, although the neonate had a prior exposure to VZV during the pregnancy, varicella did not occur in the immediate neonatal period.

In Nepal, the previous lowest reported age of HZ was at five months by Jha et al^8^ Most of the reported cases of childhood HZ acquire VZV infection from mother during intrauterine life or during delivery.^7^, ^8^ Some may acquire infection from other family members or siblings.^5^ In a study done by Vora et al, 56% of children with HZ had history of exposure to VZV.^9^ There are some cases where neither mother nor child had prior history of VZV infection before the occurrence of HZ.^10^ In our case too, neither mother nor the infant had previous history of varicella. To our knowledge, this is the first case of HZ in an infant reported in Nepal without prior history of varicella in the baby and the mother. The reason of such occurrence can be due to unrecognized subclinical varicella infection along with some occult triggering factor in infant born to varicella zoster immune mother.^8^ Such occurrences of unrecognized subclinical infection can be subjected to antibodies test to prove unexposed occurrence. Maternal VZV antibody in infant offers protection for few months but the level decreases rapidly, and after 4 months, they remain no longer protected.^11^ VZV antibodies in the mother were negative in our case whereas we could not get antibodies done in the child due to financial constraints.

Childhood HZ is slightly more common in males and generally any dermatomes can be involved,^9^ whereas in our case, the infant was female with a single dermatome (T9) involvement.

Diagnosis of HZ is generally made on clinical ground which is supported by Tzanck smear finding. Tzanck smear is not specific for HZ infection and can be suggestive of other viral infections like *Herpes simplex* virus (HSV). Although the definitive diagnosis of HZ requires polymerase chain reaction (PCR), clinical presentation of vesicular skin lesion in dermatomal distribution strongly suggests toward diagnosis of VZ whereas HSV infection is more likely to cause oral and genital lesions. Moreover, even Centers for Disease Control and Prevention (CDC) does not recommend the routine use of PCR to diagnose typical VZ and is indicated only in children with atypical presentation (immunosuppression and zoster sine herpete).^12^

There is no consensus about the use of antiviral drug in the treatment of HZ in healthy children; however, early acyclovir therapy can prevent complications. In our case, oral acyclovir was given as the presentation was early. The baby was immunocompetent and recovered well in one week without any complications.

## CONFLICT OF INTEREST

None declared.

## AUTHOR CONTRIBUTIONS

CMS, SS and NC: equally involved in drafting, literature search, and writing of the paper.

## CONSENT

Both verbal and written consents were obtained from the parents regarding the publication of the case and photographs.

## References

[1] Sterling JC . Viral infections In Griffiths CEM, Jonathan B, Tanya B, Robert C, Daniel C, eds. Rook’s Textbook of Dermatology, 9th edn Chichester, UK: John Wiley & Sons, Ltd; 2016:25.24‐25.29 .

[2] Prabhu S , Sripathi H , Gupta S , Prabhu M . Childhood herpes zoster: a clustering of ten cases. Indian J Dermatol. 2009;54(1):62‐64. 20049274 10.4103/0019-5154.48991 PMC2800875

[3] Kayastha BM , Shrestha P , Shrestha R , Lama L . Changing profile of herpes zoster in Nepal: A hospitalbased study. Nepal J Dermatol Venereol Leprol. 2009;8(1):1‐4.

[4] Nikkels AF , Nikkels‐Tassoudji N , Piérard GE . Revisiting childhood herpes zoster. Pediatr Dermatol. 2004;21(1):18‐23. 14871320 10.1111/j.0736-8046.2004.21104.x

[5] Kurlan JG , Connelly BL , Lucky AW . Herpes zoster in the first year of life following postnatal exposure to varicella‐zoster virus: four case reports and a review of infantile herpes zoster. JAMA Dermatol. 2004;140(10):1268‐1272. 10.1001/archderm.140.10.1268 15492192

[6] Smith CG , Glaser DA . Herpes zoster in childhood: case report and review of the literature. Pediatr Dermatol. 1996;13(3):226‐229. 8806124 10.1111/j.1525-1470.1996.tb01208.x

[7] Feldman GV . Herpes zoster neonatorum. Arch Dis Child. 1952;27(132):126‐127. 14924677 10.1136/adc.27.132.126 PMC1988768

[8] Jha A , Kumar A , Paudel U , Neupane S , Pokhrel DB , Badal KP . Herpes zoster in a five month old infant subsequent to intrauterine exposure to varicella infection. Nepal Med Coll J. 2007;9(4):281‐283. 18298022

[9] Vora RV , Rahul Krishna S , Kota NBJ . A clinicomorphological study of childhood herpes zoster at a rural based tertiary center, Gujarat, India. Indian J Paediatr Dermatol. 2016;17:273‐276.

[10] Jain A , Singal A , Baruah MC .Herpes zoster in a 9‐month‐old infant. Indian J Dermatol Venereol Leprol. 1999;65(6):294. 20921693

[11] Pinquier D , Gagneur A , Balu L , et al, Prevalence of anti‐varicella‐zoster virus antibodies in French infants under 15 months of age. Clin Vaccine Immunol. 2009;16(4):484‐487. 19176690 10.1128/CVI.00397-08 PMC2668269

[12] Shingles | Diagnosis, Testing, Lab Methods | Herpes Zoster | CDC. [Internet]. 2019 [cited 2020 Apr 6]. Available from: https://www.cdc.gov/shingles/hcp/diagnosis‐testing.html

